# Perceptions of Diet and Physical Activity Among California Hmong Adults and Youths

**Published:** 2007-09-15

**Authors:** Gail G Harrison, Loan Pham Kim, Marjorie Kagawa-Singer

**Affiliations:** University of California at Los Angeles Center for Health Policy Research; UCLA School of Public Health and UCLA Center for Health Policy Research, Los Angeles, California; UCLA School of Public Health and UCLA Center for Health Policy Research, Los Angeles, California

## Abstract

**Introduction:**

We conducted a qualitative study to inform the design of a proposed community-wide campaign to promote increased physical activity and fruit and vegetable consumption among low-income Hmong families.

**Methods:**

We held eight focus groups with parents of children aged 5 to 14 years and with youths aged 11 to 14 years, interviews with key informants in several Hmong communities, and interviews with professionals who conducted physical activity and nutrition activities in these communities. Sessions were tape-recorded and transcribed. We organized data using ATLAS.ti software and then analyzed the content.

**Results:**

Findings suggest that physically active lifestyles and dietary patterns emphasizing fresh foods including fruits and vegetables are valued in the Hmong culture and perceived as essential to good health. Barriers to a healthy lifestyle include limited access to safe spaces, time for adequate physical activity, access to land to grow fresh produce, and time for home preparation of food. Low incomes and marketing of unhealthy foods, particularly to children, are also problematic. Information on the healthy aspects of both traditional foods and American foods is needed in accessible formats and delivered through media and trusted community sources.

**Conclusion:**

Like other Asian groups, the majority of Hmong are first-generation immigrants. An increase in nutrition-related chronic diseases can be prevented by encouraging and reinforcing the maintenance of traditional eating patterns and active lifestyles.

## Introduction

### Rationale for the study

Almost one-third of American adults meet the criterion for obesity, and nearly two-thirds are either overweight or obese (1). The prevalence of overweight among children and adolescents has doubled since 1980 (2,3), and the rates of related chronic diseases including cancer, type 2 diabetes, and stroke and cardiovascular disease are rising dramatically (4). Improving dietary quality and increasing levels of habitual physical activity are critical public health goals for the U.S. population. The most important qualitative aspect of diet for disease prevention is adequate vegetable and fruit intake (5,6). Physical activity aids in the prevention of weight gain in addition to having other direct beneficial health effects. Evidence shows that for most adults, about 1 hour per day of moderate-intensity activity is required to prevent significant weight gain, and as little as 1 to 2 hours per week of fast walking or the equivalent significantly attenuates weight gain in middle age (6-10). Yet only about one-fourth of Americans eat the minimum recommended five servings of vegetables and fruits a day, and fewer than half of American adults participate in the recommended amount of physical activity (2,10).

Ethnic minority populations have worse health outcomes in every chronic disease category than do non-Hispanic whites. National efforts and state efforts in California provide program resources and outreach to address these disparities in African Americans and Latino populations, but no effort exists nationally or statewide in California for Asian American, Native Hawaiian/and other Pacific Islander (AA/NHOPI) communities. AA/NHOPIs are the most rapidly growing ethnic group in the United States (11,12). About 70% of AA/NHOPI adults in California are first-generation immigrants. Notably, the limited data on dietary practices and prevalence of overweight and obesity indicate that the rate of increase of obesity among AA/NHOPIs is significant. They have the fastest rate of increase in overweight and obese youths of all ethnic groups in California. California Pediatric Nutrition Surveillance System data reveal that from 1994 to 2003, overweight prevalence increased from 7% to 15% for California AA/NHOPI low-income children, more rapidly than for any other ethnic group (13). Although few data are available specifically on Hmong Americans, a local study in Wisconsin reported that 65% of Hmong youths were overweight or obese (14).

Realizing the unique opportunity to provide health promotion support to optimize dietary and physical activity practices among Asian immigrants during the all-important transition from first to second generation, California Department of Health Services and the University of California Los Angeles (UCLA) School of Public Health partnered in early 2004 to begin the formative work required to devise guidelines for an appropriate nutrition and physical activity campaign targeting Asian American (AA) communities.

Data on parallel studies among low-income Chinese and Vietnamese immigrants and their children are reported elsewhere (15).

### Hmong Americans

Unlike other groups that have a clear country of origin, the Hmong originate from several regions in Southeast Asia. A tribal people originally from China, where several million ethnic Hmong still reside, many fled to Laos to escape persecution in China. During the Vietnam War, many of them fought for the United States in Laos and were subject to North Vietnamese retribution when the United States left Vietnam. Most who survived then fled to Thailand, where they have lived in refugee camps, sometimes for many years. Most U.S. Hmong came to the United States directly from Thailand, beginning in 1979. Approximately 200,000 Hmong now live in the United States, most of whom have settled in Minnesota's Twin Cities, Wisconsin, and California's agricultural central and northern valleys. About 50% of the Hmong in the United States reside in California.

Hmong are traditionally agriculturalists, with a patriarchal and patrilocal (i.e., new families taking up residence with or near the husband's family) social structure. Religious beliefs are based in traditional animism (i.e., the belief that all elements of the earth and its creatures possess spirits or souls that live in harmony when they are satisfied that each is living with respect and honor for each other). Fertility is high (average family size is 4.5), and the average age of the population is young. The history of the Hmong language has implications for the design of health promotion programs. The written language was lost during centuries of persecution in China. Christian missionaries developed a Romanized phonetic script, which is not intuitive for pronunciation, in the late 20th century. An increasing number of materials are being printed in this Hmong rendition, but it remains difficult for most Hmong to read (16).

## Methods

We used qualitative methods to explore knowledge, attitudes, opinions, and reported behavior regarding health and healthy lifestyles, dietary practices (focused on vegetable and fruit consumption), and physical activity among low-income Hmong parents and youths in California. The work was nested in a broader study that also included first-generation, low-income Chinese and Vietnamese immigrants, reported elsewhere (15). Interview guides and inductive focus group guides were developed, and interviewers and focus group moderators were formally trained. A series of key informant interviews and focus group discussions were held. Identification of key informants, recruitment of focus group participants, and facilitation of interviews and discussions were accomplished in concert with community partners, the Hmong Women's Heritage Association in Sacramento, and Stone Soup Fresno. An advisory committee (made up of professionals and community leaders who worked extensively with the study group in the areas of nutrition and physical activity) contributed to the recruitment strategy, which can best be described as informed convenience sampling. Recruitment flyers and information sheets were distributed to our community partners, who posted the translated notices at supermarkets, businesses, community-based organizations, clinics, and schools. Key informants were identified and contacted directly by representatives from the community agencies. All materials (including interview guides, focus group guides, and recruitment materials) were translated into Hmong and back-translated to ensure integrity and consistency. Focus group moderators and interviewers were bilingual and bicultural. All aspects of the study were reviewed and approved by UCLA's Institutional Review Board for protection of human research subjects.

We interviewed five key informants in addition to 44 adults (39 women and 5 men) and 40 youths (18 boys and 22 girls) in eight focus groups in Fresno and Sacramento. Adult focus group participants ranged in age from 25 to 80 years and were parents of children aged 5 to 14 years. Youths were aged 11 to 14 years. Focus groups lasted an average of 2 hours, with question and answer sessions following, so the discourse was unhurried. Interviews and focus groups were tape-recorded and transcribed. We coded and organized the data using ATLAS.ti (ATLAS.ti Scientific Software Development GmbH, Berlin, Germany) software and then analyzed the content. The number of respondents noted in the results is lower than total participants because of the dynamics of the groups. If one person answered and the others agreed, no one else responded. Therefore, according to the focus group leaders, the lower numbers most likely represent consensus in the group.

## Results

### Definitions of health

Adult participants consistently defined health as the absence of health disorders and psychological and emotional stability, and harmony within the family. Respondents expressed a common cultural belief that being healthy meant being thin — one key informant stated that  "Hmong people are [naturally] small." Health was characterized by eating foods one desired — to be "proud of what you eat" — and eating many types of vegetables and fruits. "Balance" between consuming foods from different food groups, such as vegetables cooked with meat, was noted by adults as necessary to keep the body functioning optimally. Adults noted that cleanliness and environmental conditions contributed to overall health and emphasized showering frequently and keeping a home free of dirt and germs.

Both adults and young people recognized the health benefits of physical activity/exercise and vegetable and fruit consumption. Exercise and vegetable and fruit intake were believed to prevent health risks and illnesses such as elevated cholesterol levels, diabetes, stroke, hypertension, and weight gain. One participant expressed that "people that are not physical[ly] active seem to have more problems and mental and health problems." Specifically, low stress levels were deemed to be an essential component of health. Adults expressed worry about weight gain, diabetes, kidney disease, smoking-related cancers, and pregnancy disorders but with low frequency. Respondents commonly expressed the perception that they were not as healthy in the new environment as they were before immigration and that they faced many new health challenges.

### Perceptions about diet

Respondents generally agreed that vegetables and fruits are the healthiest types of foods and should be consumed often. Beneficial starches included rice, noodles, and bread. Meat was believed to be necessary for good health, but consumption should be limited to reasonable quantities and to lean meats, such as fish and roasted chicken, which were reported as most beneficial. Most people explained that different aspects of Hmong and American cuisine were advantageous — the high volumes and availability of vegetables and fruits in the American diet and the low levels of fatty and oily foods of the Hmong diet.

Meat was named as the single most commonly consumed unhealthy food. Many adults expressed concern that Hmong Americans eat too much meat after moving to the United States, and they eat fatty varieties, most significantly pork. Adults believed excessive volumes of meat directly caused sicknesses: "stomachache and diarrhea," "black-out," and feelings of carrying "a big load on your back." A few adults even advised that they stopped eating meat altogether or limited intake to holidays. All individuals advised that intake of fried, oily, and fast foods needed to be reduced. Adults expressed concern that consumption of these foods needs to be reduced by youths in particular. Consuming too many sweets was considered unhealthy as well.

Almost all participants reported that their diets included both Hmong and American foods, with greater proportions of Hmong foods. Rice remained a dietary staple in all home-cooked meals for Hmong Americans, complemented by reportedly high intakes of various vegetables, chicken, fish, beef, and pork. Breakfasts for children were commonly eaten at school and included cereal, milk, juice, eggs, hash browns, sausage, and fruit such as apples, oranges, and bananas. Frequently mentioned snacks were ice cream, cookies, chips, candy, soda, instant noodles, and common fruits. Youths reported that their schools served pizza, hot dogs, burritos, nachos, tacos, or hamburgers for lunch, all of which they and their parents perceived to be unhealthy. The main vegetables that they reported eating at school were salads and carrots.

Commonly eaten fruits included guava, mango, pears, pineapple, watermelon, and other melons. Home gardens contained peaches, grapes, oranges, and pears. Schools reportedly serve oranges, apples, and bananas. Youths named bananas, papayas, strawberries, and kiwi as favorites. A handful of individuals said that they missed their homeland fruits of rattan, red and gray banana, and passion fruit leaves, which are not available in the United States.

Vegetables eaten were beans, cabbage, broccoli, green beans, corn, mustard greens, carrots, peppers, eggplant, peas, squash, celery, bok choy, collard greens, and gourds. Youths expressed a liking for salad but did not distinguish the specific types of vegetables included. Participants who had home gardens grew and ate cucumber, pumpkin, corn, tomato, mustard greens, lemongrass, collard greens, and herbs including basil, green onion, cilantro, and mint. Some participants indicated they missed vegetables from their homelands such as palm and cassava leaves.

Hmong American adults repeatedly noted that fresh foods were essential to health. They value fresh foods as healthy because old, dried, frozen, preserved, or canned fruits, vegetables, and meats are believed to cause sicknesses: "Our Hmong people have diabetes and high blood pressure because we do not eat fresh meats. The meats we eat were in the freezer for a long time." Fresh foods were defined as meats from recently killed animals, recently caught fish (preferably the same day) or recently picked vegetables and fruits. They regarded fertilizer and pesticides as harmful to the nutritional value of vegetables and fruits. The universal sentiment was, "In our homeland the soils are fertile, and we don't use fertilizer to grow anything . . . so, we eat the crops and we are healthier. . . . In this country, you get sick a lot because fertilizer was used to grow all the vegetables and fruits. . . . I do not eat much anymore and I am very afraid now." In addition, temperature of food was important to the health of women, because cold water and foods "cause you to not have children" because of "abnormal period[s]." Youths voiced no similar cultural concerns.

Youths referred to diet sodas as a healthier alternative to regular sodas. All groups encouraged drinking plenty of water. Canned fruit juice and milk were also healthy drinks named. Parents often tried to eliminate or restrict soda consumption, referring to its high sugar content.

Adult participants reported not knowing the definition of a "serving" of fruit or vegetable or had never heard of the word before, whereas youths reported knowing about the concept from school or the news media. One adult reported that "Hmong people eat until their stomach is full, but food was also limited." Still another illustrated that the Hmong concept of a serving was much larger than the American serving size described in the *Dietary Guidelines for Americans*. Of adult respondents, 40% reported that youths ate five or more servings of fruits or vegetables per day; 60% of youths reported actually eating that amount or more. Almost all youth respondents (89%) said that youths should eat five or more servings daily, although none of the parents offered an opinion on how many servings of vegetables and fruits a child should eat per day.

Hmong American parents try to include vegetables in every meal to balance out the meat dishes. Fruit is consumed usually as a snack. "We don't eat fruits to fulfill our hunger, and we don't eat them with our meals." Adults noted that vegetables and fruits prolong life, protect against the sicknesses and diseases mentioned above, and provide vitamins. Youths cited that they provide energy and strength and that they "help the body grow." 

### Definitions of physical activity and exercise

Interviewees provided varying responses when asked to define the difference between exercise and physical activity. A few replied that there was no difference, but most distinguished the two as different concepts. Exercise was generally perceived as being more structured, as "using your energy" to "sweat," "make your heart pump faster," and "tighten up your muscle." As one adult participant phrased it, "Exercise is like going to a place or with a group that have machine and do to maintain your balance." On the other hand, physical activity was more broadly defined as "physically moving" and "using your strength." It most frequently included active games and sports, as well as chores and housework. A major health benefit of physical activity noted by adults and youths was prevention or improvement of health disorders. Other benefits stated were increased energy, strength and flexibility, burning of fat, keeping the body "in shape," maintaining independent living for the elderly, improved circulation, good posture, lowering of heart rate, and decreased risk of mental health problems.

Adults listed physical activities including walking, Tai Chi, stretching, cleaning house, and gardening. Traditional Hmong physical activities are farming, walking, climbing trees, hunting, fishing, swimming, cooking, and cleaning house. Traditional games include spinning tops, tag, jumping rope, kato (a very popular game in southeast Asia) and "playing house." Youths participated in housework by mowing the lawn and cleaning. Youths mostly enjoyed sports as their form of exercise, including basketball, volleyball, soccer, kato, baseball, running, football, tennis, badminton, and kickball. Dancing was also popular.

The average response of the five youth and adult participants who answered the question about frequency of physical activity among youths was 3.2 times per week. Sixty percent of youths and 66% of adults thought that young people get at least an hour of physical activity per day. Sixty percent of youths and 13% of adults felt that children should get an hour of physical activity each day. Most adult respondents said they participated in physical activity or brought their children to parks for physical activity. Two replied that they had enough space in their homes for their children to be physically active. Some indicated that they walked around the neighborhood streets. Most youths reported that they engaged in physical activity at a park or at school in physical education classes, during class breaks, or after school. Participants indicated that schools offer most of the physical activity venues and opportunities available to children, and a few participants mentioned churches initiating physical activity programs. Two parent participants cited that homework limited the amount of time available to youths for physical activity. Parents and youths reported that youth dedicated several hours per day (average 2.2 hrs) to watching television, using the computer, or playing video games. Half reported greater time spent using the television, computer, or video games during weekends, while the other half reported less time because they increased physical activity during weekends.

### Concern about obesity

Many parents expressed concern for their children and themselves about becoming obese after moving to the United States. The cause was perceived to be increased availability and intake of food, increased consumption of fatty American foods such as fast foods and fatty meats, and decreased physical activity levels. Before coming to the United States, "Hmong used to work on farms from morning to afternoon. They exercised all day long. In America, things are different. We are more sedentary and do not exercise as much." One gentleman cited an apt understanding of the phenomenon: "For example, like a pig. If we caged this pig then he will be fatter than the one that you let it run around. Now we are like this pig that [is] in the cage."

### Barriers to healthy living

Regarding the traditional Hmong diet, all five key informants reported changes in the traditional Hmong American diet as this community has adapted to American life and adopted some unhealthy eating habits. Although most Hmong Americans grew their own vegetables and fruits in Laos, many cannot do so in the United States because they live in small apartment complexes and no longer have their own farm or large plots of land on which to plant foods and raise animals. As a result, people do not eat as many fresh foods, which Hmong Americans define as healthy foods, as noted above. One parent reported, "Back in the homeland, there were no fertilizers used on our foods because we grew our own foods so they were healthy for our body. In this country, the foods grow because of fertilizer and pesticide use and that affects our being unhealthy." 

Although some Hmong American families in Fresno and Sacramento maintain small gardens in their backyards, the variety of traditional vegetables and fruits they are able to grow in the United States is less than those grown in Laos. In addition, concern about the presence of chemicals in produce was expressed by many participants.

One parent cited shifts in dietary practices involving eating more meat: "We were able to eat healthy foods in our homeland, but we didn't have as much foods available. We can eat more foods in this country because they're plentiful. . . . For example, there's more meat such as pork here and people tend to eat more of it." Other parents agreed that they also eat bigger portions of food here than they did in their homelands. One parent noted, "We have changed a lot. In this country, some families, every month they will kill a pig and eat for the whole month. In our homeland, we only get to eat meat once or twice each year." Although all key informants noted that it is easy to buy many different kinds of vegetables and fruits at the Asian grocery stores, some Hmong Americans are not eating as many vegetables and fruits because of lack of time and money to purchase the vegetables and fruits with which to prepare traditional meals with fresh ingredients.

Because many Hmong American households have low incomes and both parents are employed, time and money are two of the greatest barriers to eating healthy foods. Although vegetables and fruits are readily available, many Hmong American families increasingly are consuming poorer-quality diets including fast foods, fatty meats, and ready-made, frozen, and preserved foods because these options are readily available and often require less time to prepare and less money to purchase. One mother said, "We eat outside when I am tired or do not feel like cooking. Sometimes I don't have time to cook." Another parent stated, "We eat mostly Hmong food. . . . But if we don't have time then we will eat American foods. . . . The children like to eat fast food, like frozen food that is ready to serve after you microwave because they don't have the patience to wait for Hmong foods."

Many families use food stamps, which are often saved to buy meat. One parent revealed that with big families, the food stamps sometimes are not enough to purchase the needed groceries: "The more children you have, the tighter your money is when it comes to buying groceries. . . . You also worry about food when you have guests come over. There are times you have to use cash to help pay for groceries along with food stamps." One parent summed up the financial hardships:

The things that can change the way we eat would be employment and earning a higher income, which would allow us to purchase better foods. It's hard to eat healthy when you don't have a lot of money. And when you have financial limitations, you tend to purchase foods that are cheaper and unhealthy.

### Barriers to physical activity and exercise

All key informants cited lack of time and money as the major barriers to physical activity and exercise for the Hmong American community. One key informant stated, "The parent needs to supervise their kids and sometimes they don't have the time. . . . Money is a problem if you have to pay to have their children in a program." Another key informant similarly mentioned time and money issues and added that parents are sometimes not knowledgeable about what kinds of physical activity programs are available for their children:

Parents are not informed about organized youth activities programs. To participate in sport activity one will need money, and many Hmong people don't have much money. They also have lots of children. In addition, they do not have time after school to attend to all the children's sport activities.

Other parents agreed when one mentioned that language barriers hinder parents from knowing about physical activities programs for their children: "It is hard in this country. Many people do not speak English so it's hard for them to join programs." Another parent expanded on this issue:

The difficult thing is the age of parents and the language barrier. You don't know the directions to take your kids to recreational places. It's difficult to communicate with people you meet there. Also, your children may want to travel out of town but you're old and you can't drive far even if you want to. But we do like to be with our kids when we go to nearby recreational places.

In addition to language barriers, lack of information, time, and money, all key informants cited safety issues as a barrier to physical activity and exercise. One key informant noted:

The majority of Hmongs live in apartments. They tend to stay inside. Instead of exercise, they tend to take the car. Some areas where the Hmong live, it is not safe to be outside. They are afraid of gangs and shootings.

Some parents reported prohibiting their children from joining sports activities because they do not want their kids to "fool around" and forget their studies or play away from home too much for fear that playing in groups in some public areas may lead to encounters with gangs. All the parents agreed that being physically active is good for children, but they also agreed when one person added:

Yes. But their physical activities should not take up too much of their time that it has a negative impact on their studies. And you as a parent should not give them full freedom as to being physically active. They may be involved with the wrong group of kids.

Another barrier to physical activity and exercise is the lack of space and organized sports programs. One key informant revealed that, "In the south side of Fresno, there are not many places to exercise. Safety is an issue for many parents. Parents don't have time, so it's hard to pick their children up from places like the Boys and Girls Club."

Some youth focus group participants also mentioned safety as a barrier, saying, "I don't feel safe around the neighborhood." Another mentioned that there is "no space to play and they [the parents] do not let us play in the street."

Many parents also cited the lack of free public parks and public space where they can take their children to play:

As much as we'd like our children to be physically active, sometimes it costs money to enter some parks and that keeps us from taking our children. To encourage them to be physically active, it costs money to get them into the park. Our Hmong children don't get enough physical activity compared to American children because we face limited money and space.

A few parents were worried that their children, especially their daughters, might get injured ("break their leg or arm") or do harm to their bodies if they played too much sports. One parent stated, "If the girls play too much, it's not good for them because when their body is hot and they drink cold water. . . that will cause them to have abnormal periods." Some youth focus group participants also expressed that fear of getting injured, or actually being injured, prevented them from doing some sports activities.

### Ideas to encourage and maintain vegetable and fruit consumption

All key informants and parents believed that it is important to involve the whole community in any efforts to encourage healthy lifestyles for children. Foremost is involving the family and parents and educating them about healthy eating practices so that they, in turn, can maintain their own healthy habits as well as learn new ways to encourage their children to eat more vegetables and fruits. One key informant stated:

I think parents are the key. They need to be educated so they can teach their kids. Parents need to understand the difference between healthy foods and junk foods. . . . The parents need to be educated about proper nutrition. Parents are the ones that buy the food. I tried not to buy cookies and soda for my kids.

The parents agreed with this assessment when asked what would encourage their children to eat five or more vegetables and fruits each day. One parent replied, "I think for the younger children they probably don't eat, but I think for the older children if you buy and have it at home then they will eat." Another parent revealed how she encourages her daughter to eat more vegetables and fruits when she shared, "I told her that this is how we always eat. She has to eat this to help her body, so that she doesn't have diabetes and high blood pressure." This message of eating vegetables and fruits for a healthy body was emphasized by many parents. One focus group participant shared, "I tell my child, 'Eat vegetables so you'll be strong,' then he eats it. When it comes to fruits, if you the parent eats it then they will see it and want to eat it also."

Parent modeling of healthy behaviors for children was frequently mentioned. One parent stated:

The parent has to be the one eating the fruits to model for their children. Young children will eat fruits when they see parents eating [them]. For example, the parent eats a banana for breakfast while the young child eats half a banana. Also, when you eat an apple, you cut it into slices and give it to your child. To encourage your children to eat fruits, you have to purchase a variety of fruits and make them available in the home. If you let your child decide [whether to eat fruits], your child will choose not to eat fruits.

The key informants concurred with this idea and suggested several ways to encourage eating more vegetables and fruits: "The Hmong depend on their family. Teaching the parents is very important. Not all Hmong know where to go to get health information. Nonprofit organizations are a good way to get information out. The health department is good also. Social events and passing out flyers." 

Many parents agreed that receiving information from community and clan leaders, as well as from nonprofit organizations, doctors, and teachers would encourage them to pay more attention to healthy eating practices, including eating more vegetables and fruits. Notably, parents expressed interest in participating in educational workshops conducted by community health workers, doctors, and teachers.

Youth focus group participants also mentioned that they would be more likely to eat more vegetables and fruits if their parents (especially the mothers, because they are the ones who often prepare the meals) bought them and encouraged them to eat them: "Cut [up] fresh, not old fruits or vegetables. If it smells good, then it will make you want to eat more." Other youths reported that they would want to eat five or more vegetables and fruits each day if they wanted to be healthier or "to lose weight or go on a diet." Others reported that they would eat more vegetables and fruits "if we can eat it watching TV," or "eat with friends." 

### Ideas to encourage and maintain physical activity

Similar to encouraging healthy eating practices, parental involvement is an important factor in encouraging children to be physically active. Many parents reported that although they don't have much time, they do make an effort to play with their children around the house and neighborhood or take their children to nearby parks or public recreational areas. Playing with them also allows the parents to supervise their children. One parent stressed:

As a mother, you should go with children to the parks in order to supervise their activities so they don't face problems that may arise. For example, when you're with your children, they're less likely to get involved with at-risk youth groups and wander off and get into trouble.

Another parent suggested, "We need more safe places for family to enjoy themselves — like safe parks." Others also suggested having free after-school programs supervised by teachers or volunteers. Many parents noted that children are more likely to engage in exercise and physical activities when they can do so with other children. One parent explained that, "For those who live in apartment complexes like ours, the children are always outside and engaged in physical activities with other children living in the same complex. Children are more likely to do physical activities with other children and in a group."

The youth focus group participants also reported that family encouragement and involvement (e.g., going to the park together and "doing fun things," or "do exercise with your family") would help add more physical activity to their daily life. They also reported that having school programs, tournaments, and community services would encourage them to exercise and to be more physically active, especially if the activities are interesting and fun and done with their friends. Many of the youth participants understood the health benefits of exercising (e.g., "be in good shape," "have more energy," "it burns your calories," "it burns fat," "if you don't want to be fat just do exercise") and said that they would emphasize these positive aspects in telling their friends about the importance of doing at least 60 minutes of physical activity each day. Peer pressure and wanting to be thinner were other factors that the youth reported as motivators to be more physically active.

### Specific strategies and suggestions for information dissemination

#### Media, television, and radio

All key informants and focus group participants cited the media, especially Hmong-language radio and television stations, as the best channel for health information dissemination. One parent elaborated: "The news is very important in the sense that it has the ability to reach every family's home. News provided through radio announcements and on television. We always watch and listen to the news, especially the Hmong-language radio stations." Several local radio stations and television channels broadcasting in Hmong language were cited, but cartoon channels and the Public Broadcasting Service  also received mention.

Most respondents felt that newspapers would not be as good a media channel for information dissemination because there are few Hmong-language newspapers and some Hmong Americans, especially older adults, cannot read English or Hmong. One parent reminded the group:

You have to remember that many Hmong cannot read and write in their own language. In addition, there will be a new group of Hmong coming to Fresno. These Hmong might speak Thai. I think videos that are visual will be good. Radio is also a good way to outreach. Radio talk show and radio public service announcements are good. We have to outreach to the parents in a way that they can understand.

#### Culturally appropriate educational materials

Most participants continually emphasized that any educational materials or media campaigns should use a lot of visual and audio aids instead of written materials because many Hmong Americans cannot read. Many parents expressed the desire to attend more educational workshops or classes on nutrition and physical activity. They wished that more classes were offered in the community because they wanted to learn more about these topics. One parent mentioned:

We need educational classes in which the information can be translated to our language. The use of visual aids is also important, such as having the food samples and serving sizes. It's also good to have both visual and audio material available to us. It's important to have someone that's educated about the nutrition and health field to teach us parents about eating and leading a healthy lifestyle. Once we're educated we can help our children to eat healthier. Health education classes are important and the curriculum should address the specific foods and their nutritional benefits.

In addition, parents suggested, "We can learn from visual aids like posters and pamphlets, which should be taught and explained by an educated professional." Many also suggested having health videos. One parent emphasized, "It's important that the visual aids [be in] the Hmong language so that the older folks can read it and understand it." Other educational materials suggested by the youths included magnets, book covers, stickers, key chains, flyers, and posters. The youths preferred to have materials with lots of pictures.

The most popular locations for educational workshops and outreach included Asian grocery stores, nonprofit agencies, community-based social service organizations, and health clinics with Hmong-speaking staff. Key informants and parents also suggested holding educational outreach events at churches, New Year's festivals, health fairs, and flea markets.

When asked if they had ever seen the 5 A Day logo, most adults reported that they had not. The key informants mentioned that they had seen the logo in American supermarkets, but not in the smaller Asian grocery stores. Many of the youth focus group respondents, on the other hand, did recognize the logo. They indicated that they saw the logo on posters at school.

#### Health information messengers

The youth focus group participants most frequently mentioned teachers and mothers as the people from whom they get information about eating healthfully and exercising. Hmong-speaking doctors, community health workers, and community leaders were frequently mentioned by the adult respondents as people from whom they would like to receive educational health information. However, one parent mentioned that the educators did not necessarily have to be Hmong, because "[t]he ethnicity of the person distributing or teaching the information does not matter to us, but it is important that the person be educated" in the area he or she is teaching.

Trust is an important factor in selecting the messenger for health information. One parent articulated this crucial point by saying:

I believe in doctors and educated individuals. One concern about the distribution of information is trust. In the past, Hmong people have been given inaccurate and wrong information about various things (such as life insurance), and in some ways, they've lost trust in those educated individuals. The best way to distribute information would be . . . agencies . . . that have cultivated a trustworthy reputation among the Hmong community.

## Discussion

The traditional diet of first-generation Hmong Americans includes many fresh vegetables and fruits, and the health benefits of physically active lifestyles are valued and clearly perceived. Study participants articulated the barriers they face to maintain these healthy lifestyles in the United States and felt that overweight and ill health are predictable results. These findings are consistent with other reports of Hmong cultural models of health. For example, Culhane-Pera et al (17) describe Hmong perceptions of diabetes causation in the terms of their sense that "we are out of balance here."

Several themes emerged from the information we collected:

- The general concept of health and healthy lifestyles not only involves eating healthful and nutritious foods and being physically active but also prominently includes both individual mental and emotional health and family harmony.
- The concept of "balance" is fundamental to the concept of health: mental, emotional, physical, interpersonal, and metaphysical.
- The idea of "freshness" is directly related to perceptions of healthful foods. "Fresh" foods are not frozen, dried, canned, or preserved and are pesticide- and hormone-free. In the case of meat and fish, recently slaughtered or caught (same day if possible) items are deemed fresh.
- Physical activity and exercise are generally understood and believed to be beneficial to the overall health of individuals through increasing energy and strength, improving physical and mental well-being, promoting weight loss, and preventing illness. Physical activity is perceived to include not only common sports and games but also cooking, household chores, and yard work.
- There was a clear theme of parental responsibility for children's lifestyles and for modeling healthy behaviors.

The longer the Hmong live in the United States, the more their diet inevitably will change to include more of the foods most commonly eaten in North America. Parents in our study clearly understood what would be healthier choices, but lack of time, money, and knowledge about the value-maintaining aspects of the traditional Hmong diet are all constraining factors. In terms of physical activity, lack of time, money, and available safe spaces to be active are the most important perceived barriers. The articulated need for better education and better jobs to enable them to overcome these barriers is clear.

In addition to individual and social constraints on making healthy choices about diet and physical activity, changes in the environment — nutritional, social, and environmental — are also necessary.  Parents and youths reported that the meals provided for children at school are neither tasty nor healthy. While the demands of schoolwork and the availability of television, video games, and computers compete with time devoted to physical activity, safety concerns and limited access to parks and playgrounds are also preventing children from getting enough daily physical activity.

There is a clear opportunity to help communities of first-generation immigrants avoid developing increased burdens of nutrition-related chronic disease through providing information and addressing structural and environmental barriers to physical activity and optimal diets. Barriers to optimal diets and physical activity patterns for the Hmong community are multilevel, and a socioecological framework is therefore essential as a basis for designing effective interventions.

This study was a small qualitative exploration of current knowledge, attitudes, and perceptions about vegetable and fruit consumption (and by inference, diet in general) and physical activity among low-income, first-generation Hmong Americans and their families in California. There were several limitations to this study. The study participants were volunteers. The results are not representative of the populations from which the participants were drawn and cannot be generalized to other Hmong Americans. Despite these limitations, this study is among the first to document the knowledge, attitudes, and practices in the Hmong community in California on these topics.

## References

[1] Flegal KM, Carroll MD, Ogden CL, Johnson CL (2002). Prevalence and trends in obesity among US adults, 1999–2000. JAMA.

[2] Ogden CL, Flegal KM, Carroll MD, Johnson CL (2002). Prevalence and trends in overweight among US children and adolescents, 1999–2000. JAMA.

[3] Hedley AA, Ogden CL, Johnson CL, Carroll MD, Curtin LR, Flegal KM (2004). Prevalence of overweight and obesity among US children, adolescents and adults, 1999-2002. JAMA.

[4] (1997). World Cancer Research Fund, American Institute for Cancer Research. Food, nutrition and the prevention of cancer: a global perspective.

[5] Knoops KT, de Groot LC, Kromhout D, Perrin AE, Moreiras-Varela O, Menotti A (2004). Mediterranean diet, lifestyle factors, and 10-year mortality in elderly European men and women: the HALE project.. JAMA.

[6] Fogelholm M, Kukkonen-Harjula K (2000). Does physical activity prevent weight gain — a systematic review. Obes Rev.

[7] Erlichman J, Kerbey AL, James WP (2002). Physical activity and its impact on health outcomes. Paper 2: prevention of unhealthy weight gain and obesity by physical activity: an analysis of the evidence. Obes Rev.

[8] Saris WH, Blair SN, van Baak MA, Eaton SB, Davies PS, Di Pietro L (2003). How much physical activity is enough to prevent unhealthy weight gain? Outcome of the IASO 1st Stock Conference and consensus statement. Obes Rev.

[9] Littman AJ, Kristal AR, White E (2005). Effects of physical activity intensity, frequency, and activity type on 10-y weight change in middle-aged men and women. Int J Obes (Lond).

[10] (2002). Institute of Medicine. Dietary reference intakes for energy, carbohydrates, fiber, fat, fatty acids, cholesterol, protein and amino acids.

[11] (2002). U.S. Census Bureau. The Asian population: 2000.

[12] Lai E, Arguelles D (2003). The new face of Asian Pacific America: numbers, diversity, and change in the 21st century.

[13] (2006). California Pediatric Nutrition Surveillance System, 1994–2003 data.

[14] (2001). Result of Hmong health screenings.

[15] Harrison GG, Kagawa-Singer M, Foerster SB, Lee H, Pham Kim L, Nguyen TU (2005). Seizing the moment: California's opportunity to prevent nutrition-related health disparities in low-income Asian American populations. Cancer.

[16] The Hmong in America: a story of tragedy and hope.

[17] Culhane-Pera KA, Her C, Her B (2007). "We are out of balance here": a Hmong cultural model of diabetes. J Immigr Minor Health.

